# The protective effect of Ozone on the mice testicular damage induced by methotrexate

**DOI:** 10.5935/1518-0557.20240041

**Published:** 2024

**Authors:** Layasadat Khorsandi, Negar Varaa, Reza Dadfar, Sadegh Moradi Vastegani, Asadi-Fard Yousef, Akram Ahangarpour, Amirhesam Keshavarz-Zarjani

**Affiliations:** 1 Cellular and Molecular Research Center, Medical Basic Sciences Research Institute, Ahvaz Jundishapur University of Medical Sciences, Ahvaz, Iran; 2 Department of Anatomical Sciences, Faculty of Medicine, Ahvaz Jundishapur University of Medical Sciences, Ahvaz, Iran; 3 Department of Anatomical Sciences, Faculty of Medicine, Fasa University of Medical Sciences, Fasa, Iran; 4 Department of Anatomical Sciences, Faculty of Medicine, AJA University of Medical Sciences, Tehran, Iran; 5 Department of physiology, faculty of medicine, physiology research center, Ahvaz jundishapur university of Medical Sciences, Ahvaz, Iran; 6 Department of Anatomical Sciences, Faculty of Medicine, Arak University of Medical Sciences, Arak, Iran; 7 Student Research Committee, Ahvaz Jundishapur University of Medical Sciences, Ahvaz, Iran

**Keywords:** ozone, methotrexate, chemotherapy, oxidative stress, testis

## Abstract

**Objective:**

Methotrexate (MTX) is widely administered for the treatment of various cancers. However, MTX induces male reproductive toxicity. In the current study, the effect of ozone therapy (OT) on reducing the toxic effects of MTX in the mouse testicles has been investigated.

**Methods:**

Twenty-four mice were divided into four groups: control, OT (4 mg/kg ozone), MTX (20 mg/kg), and MTX + OT. Testosterone levels, histological changes, and oxidative stress biomarkers were assessed to evaluate the protective effects of OT.

**Results:**

The results demonstrated that MTX disrupted germinal epithelium, reduced serum testosterone levels, and enhanced oxidative stress in testicular tissue. However, treatment with OT attenuated these adverse effects. OT effectively restored the levels of antioxidant enzymes, such as catalase (CAT), glutathione (GSH), and superoxide dismutase (SOD). OT reduced lipid peroxidation, as indicated by decreased malondialdehyde (MDA) levels. OT preserved normal spermatogenesis, improved morphometric parameters, and reduced histological changes by MTX. Moreover, OT effectively restored testosterone levels.

**Conclusions:**

OT protects against MTX-induced testicular damage by suppressing oxidative stress.

## INTRODUCTION

Chemotherapy is a practical choice to treat cancer (Mathan *et al.,* 2022). Methotrexate (MTX) is a chemotherapeutic agent for managing malignancies, such as acute lymphoblastic leukemia, lymphoma, and breast cancer (Yulug *et al.,* 2013). Many studies indicated that MTX has toxic impacts on seminiferous tubules, impairs spermatogenesis, and induces sperm DNA mutation in mice (Padmanabhan *et al.,* 2009) or rats (Daggulli *et al.,* 2014; Kilinc & Uz, 2021). These events are caused by heightened levels of reactive oxygen species (ROS) induced by MTX (Yulug *et al.,* 2013). Since oxidative stress promotes male infertility, antioxidants can improve fertility (De Luca *et al.,* 2021). Previous studies demonstrated that ozone therapy (OT) can potentially decline pathological complications caused by oxidative stress (Al-Gendy & El-Sharkawy, 2016; Tusat *et al.,* 2017).

The mechanism responsible for the activation of antioxidant cascades by OT is the formation of hydrogen peroxide and ROS, as well as malondialdehyde (MDA). Then this slight oxidative stress induced by OT induces catalase (CAT), superoxide dismutase (SOD), and glutathione (GSH) generation (Bocci *et al.,* 1998; Inal *et al.,* 2011; Merhi *et al.,* 2018). OT improves sperm quality and reduces oxidative stress in testicular disorders induced by chemotherapy drugs, testicular torsion, and estrogen-induced testicular toxicity (Merhi *et al.,* 2018; Mills *et al.,* 2015; Tusat *et al.,* 2017). This study explores whether OT effectively protects the mouse testis against MTX-induced testicular tissue toxicity by evaluating oxidative stress.

## MATERIAL AND METHODS

### Animals and Experimental Design

Twenty-eight NMRI mice (6-8 weeks; 25-30 g) were used in this work. This study was done in the animal house of the Cellular and Molecular Research Center, Ahvaz Jundishapur University of Medical Sciences, Ahvaz, Iran. The mice were kept under the same standard conditions, which included 12 hours light/ 12 hours darkness, 25°C with free access to water and food. This work was done with the approval of the Ethics Committee of Jundishapur University of Medical Sciences, Ahvaz (IR.AJUMS.ABHC. REC.1401-016). The study groups were the following groups (seven animals per group).

- Control group: 0.2 ml normal saline (i.p.) for ten days.

- OT Group: 4mg/kg OT intraperitoneally for ten days.

- MTX Group: Normal saline (i.p.) was administered for ten days, and a single dose of MTX (20 mg/kg) was injected on the seventh day

- MTX + OT Group: OT was administered for ten days, and a single dose of MTX (20 mg/kg) was injected on the seventh day (Yulug *et al.,* 2013).

Ozone has been generated by an oxygen-to-ozone converter (Gardina Co.) that produces a mixture containing approximately 3% ozone/oxygen. Ozone concentration has been determined by ultraviolet light with a wavelength of 254 nm.

On the 11^th^ day, blood samples were collected under deep anesthesia, the left testis was fixed in Buin’s solution for histological examination, and the right was frozen to measure the levels of CAT, GSH, SOD, and MDA.

### Testosterone measurement

The blood samples were collected directly from the hearts under deep anesthesia. After clotting, the serum was separated by a centrifuge (200 rpm, 15 min). A mouse ELISA testosterone kit (ARG80662, Taiwan) was used to measure testosterone serum levels.

### Histological study

The right testes of mice were fixed, and histological slides were prepared. The slides were stained with Hematoxylin and Eosin (H&E), and structural changes were assessed under a light microscope. The number of seminiferous that had vacuoles was divided by the number of healthy tubes in a field and multiplied by 100. At least 20 fields were examined for each testis.

Johnsen’s testicular biopsy score was used to evaluate the maturation of spermatogenesis. In brief, After counting more than 100 cross-sectioned somniferous tubules per/ animal, they were ranked from 1 to 10, as previously described (Johnsen, 1970).

The seminiferous tubule diameter was determined by measuring the distance between two basal membranes at two opposing poles using the Motic Images software program at 400 x magnification.

### Measuring MDA, SOD, CAT, and GSH

To measure the oxidative stress biomarkers, the left testicles of mice were kept in a -80 freezer. The tissues had homogenized, and MDA, SOD, CAT, and GSH were measured using ZellBio GmbH (Germany) kits.

### Statistical analyses

The sample size calculated by Power and Sample Size Calculation Software (version 3.1.2) was 28 male mice. The statistical power of the study was 85%. Statistical analyses were conducted using SPSS version 21.0 (SPSS, Chicago, IL, USA). ANOVA and the post-hoc least significant difference (LSD) or Tukey’s tests were performed to multiple comparison analyses to assess differences among groups. A *p*<0.05 was assumed significant.

## RESULTS

### Testosterone assay

**Figure 1:**
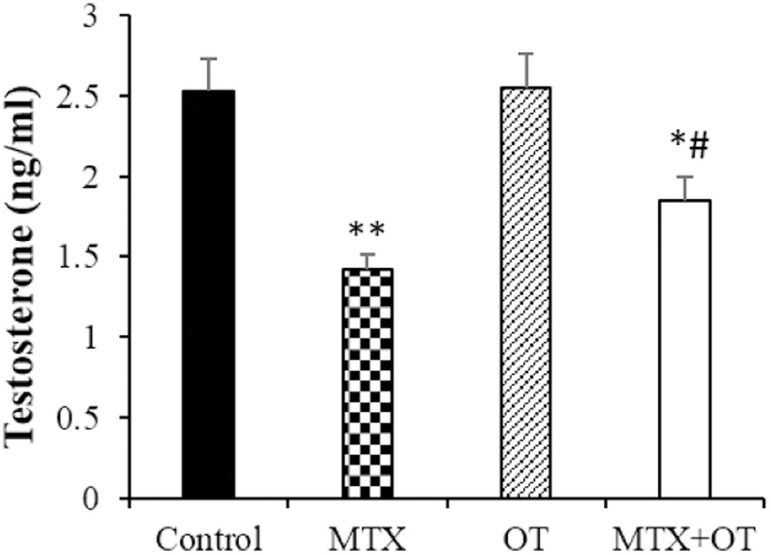
Testosterone levels in the different groups (Mean ± SD; n = 7). * & # *p*<0.05, ** *p*<0.001. The asterisk and # symbols indicate a comparison to the control and MTX-intoxicated groups, respectively.

There was no difference in testosterone concentration between the control and OT groups. A significant decrease in this hormone concentration occurred in the MTX group compared to the control (*p*<0.001). In the MTX+OT group, the testosterone level was increased compared to the MTX-injected mice (*p*<0.01) (Figure 1).

### Histology

**Figure 2:**
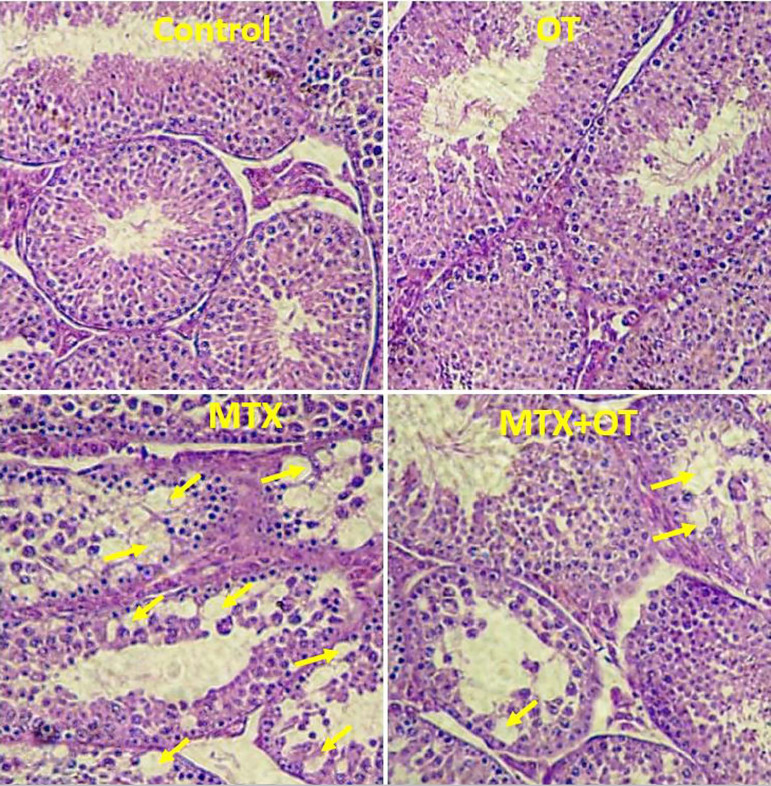
Light microscopy of testicular tissue from the control and experimental groups (Arrows show vacuoles in the germinal epithelium); H&E staining; Magnifications: ×250.

**Figure 3:**
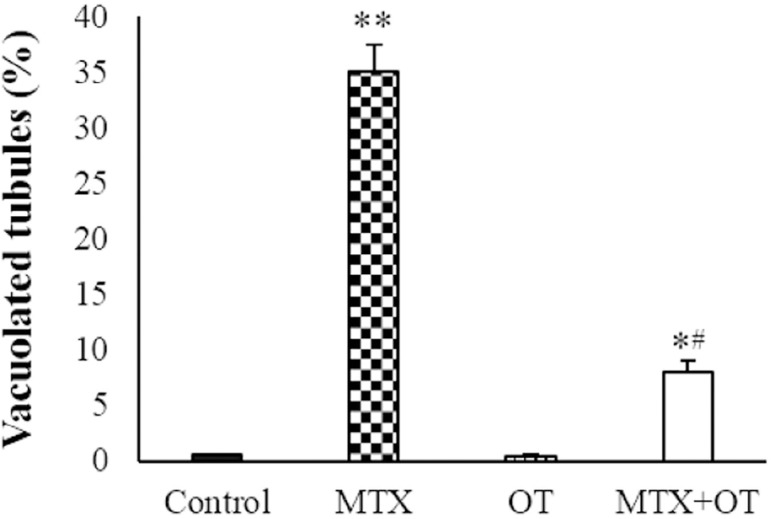
Testosterone levels in the different groups (Mean ± SD; n = 7). * & # *p*<0.05, ** *p*<0.001. The asterisk and # symbols indicate a comparison to the control and MTX-intoxicated groups, respectively.

The cross sections of the testicular tissue (Figure 2) showed normal spermatogenesis and intact epithelium of seminiferous tubules in the control and OT groups. In the MTX group, the percentage of vacuolated seminiferous tubules was significantly increased compared to the control (*p*<0.001). The vacuolization was lesser in the MTX+OT group than in the MTX-treated animals (*p*<0.01, Figure 3).

**Figure 4:**
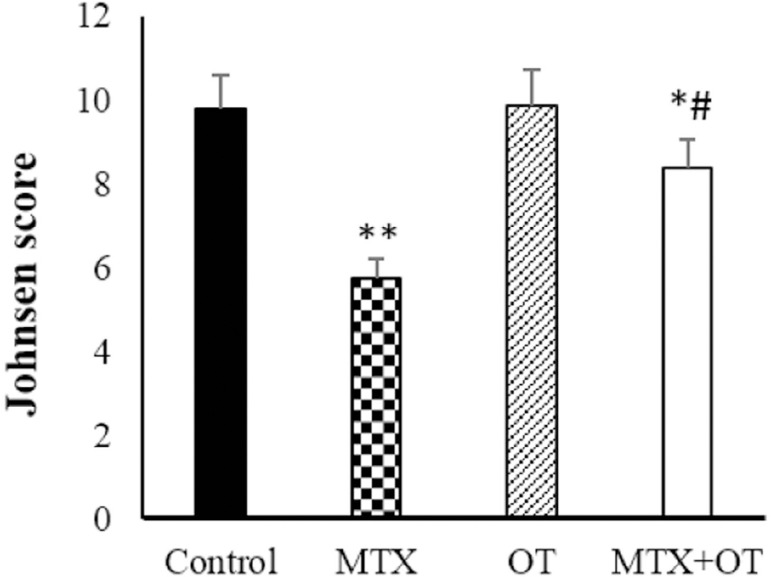
Johnsen scored assessments in the different groups. Values expressed as Mean ± SD for six mice. * & # *p*<0.05, ** *p*<0.001. The asterisk and # symbols indicate a comparison to the control and MTX-intoxicated groups, respectively.

Assessment of spermatogenesis maturation based on the Johnsen score (Figure 4) demonstrated a significant diminish in the MTX group compared to the control (*p*<0.001). However, Johansen’s scoring in the MTX+OT group increased compared to the MTX alone (*p*<0.001).

### Morphometry parameters

**Figure 5:**
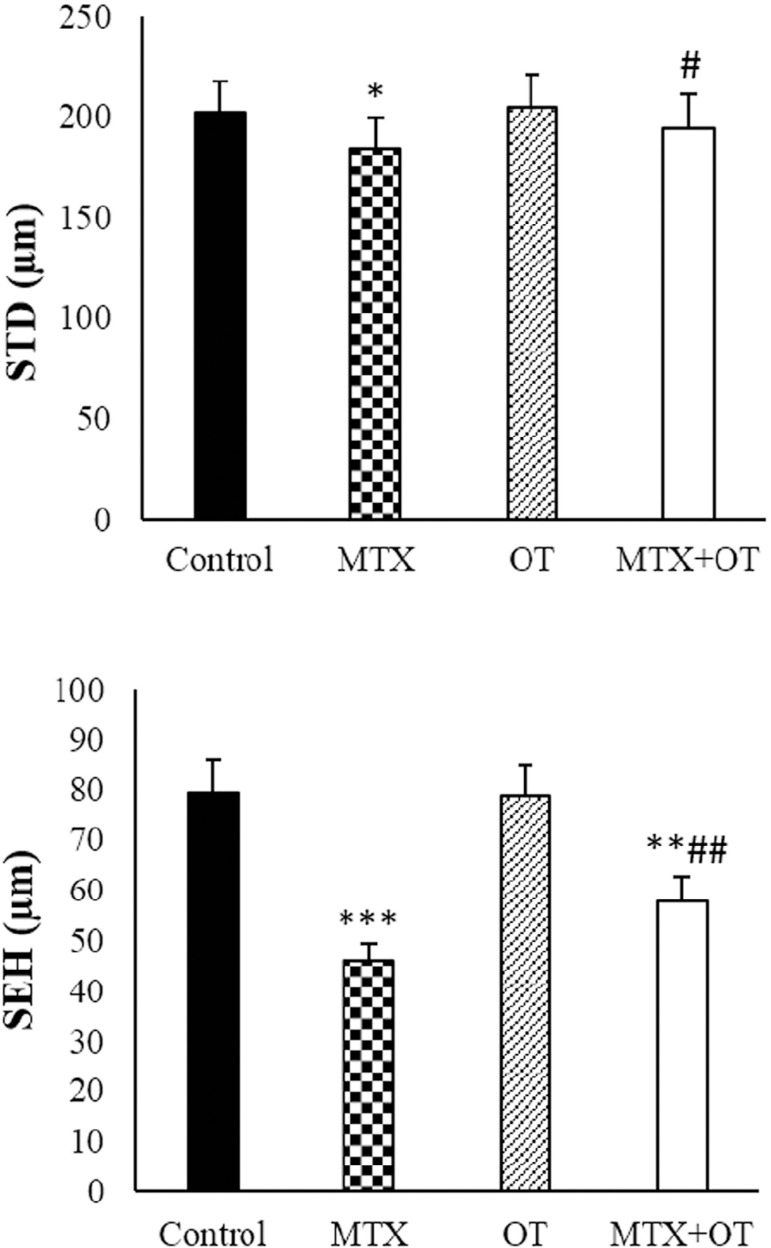
Seminiferous tubule diameter (STD) and seminiferous epithelium height (SEH) in the different groups. Values expressed as Mean ± SD for six mice. *& # *p*<0.05, **& ## *p*<0.01, ***p<0.001. The asterisk and # symbols indicate a comparison to the control and MTX-intoxicated groups, respectively.

As depicted in (Figure 5), the OT and control groups had similar seminiferous epithelium height and seminiferous tubule diameter. Morphometric parameters significantly diminished in the MTX-injected mics. In the MTX+OT group, morphometric parameters were significantly elevated compared with MTX-injected animals (*p*<0.01).

### Oxidative stress biomarkers

**Figure 6:**
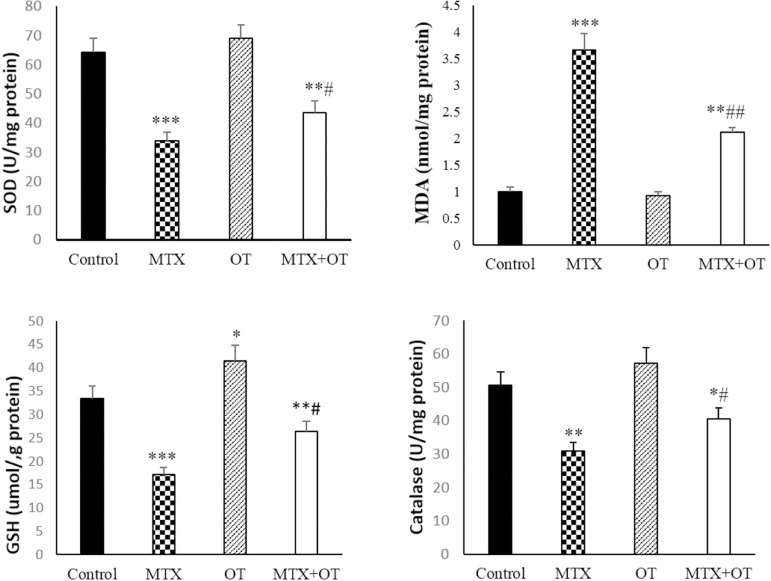
Oxidative stress biomarkers in different groups (Mean ± SD; n = 7). *&#*p*<0.05, **&##*p*<0.01, ***p<0.001. The asterisk and # symbols indicate a comparison to the control and MTX-intoxicated groups, respectively.

In the MTX group, the amount of MDA increased compared to the control (*p*<0.001). MDA level in the MTX+OT group significantly decreased compared to MTX (*p*<0.001). SOD and CAT levels decreased in the MTX group compared to the control (*p*<0.001). In the MTX+OT group, the amount of CAT enhanced compared to MTX alone (*p*<0.05), however, it was not significant for SOD. The amount of GSH was elevated in the OT group compared to the control group (*p*<0.001). GSH decreased significantly in the MTX group compared to the control (*p*<0.001). GSH levels were significantly elevated in the MTX+OT group compared to the MTX-treated animals (*p*<0.01). These results are available in the (Figure 6).

## DISCUSSION

In this study, MTX disrupted the germinal epithelium, reduced serum testosterone, and induced oxidative stress in the testicular tissue of the mice. Many studies have been implemented about chemotherapy drugs and their destructive effects on body tissues. One of the most widely used chemotherapy drugs is MTX, which causes oxidative stress and induces reproductive toxicity (Daggulli *et al.,* 2014; Yüncü *et al.,* 2015).

The decreased activity of SOD and CAT enzymes caused by the MTX is in line with other studies (Belhan *et al.,* 2019; Maremanda & Jena, 2017; Vardi *et al.,* 2009). SOD is one of the primary antioxidants in spermatogenesis, which protects the testicular tissue against oxidative stress (Fujii *et al.,* 2003; Yaman *et al.,* 2018). Cellular exposure to MTX enhances susceptibility to oxidative stress, attributed to a reduction in the quantity of free NADPH, a crucial cofactor utilized by GSH. Thus, cells become vulnerable to ROS-related damage when the antioxidant defense system is markedly diminished in the MTX exposure (Vardi *et al.,* 2009). OT could partially decrease SOD levels, significantly increase CAT activity and GSH contents in the MTX-intoxicated mouse testicles. These findings indicate that OT activates both enzymatic and non-enzymatic antioxidant systems.

In the current study, MTX could also significantly increase the amount of MDA in the testicular tissue, consistent with previous studies (Pinar *et al.,* 2018; Yalcin *et al.,* 2020). MDA, a product of lipid peroxidation, indicates tissue damage induced by MTX (Belhan *et al.,* 2019; Yaman *et al.,* 2018). MDA levels in the testicular tissue of the MTX+OT treatment have reversed, which indicates that OT can attenuate lipid peroxidation in the mouse testicular tissue. In line with our results, OT reduced lipid peroxidation in the testicular tissue of Busulfan-treated mice (Moghadam *et al.,* 2021).

Ozone may induce moderate oxidative stress due to interaction with intracellular elements. Moderate oxidative stress enhances Nrf2 (nuclear factor-erythroid 2-related factor 2) transcription. Nrf2 activates antioxidant response elements. Activating these elements results in the generation of several antioxidant enzymes, such as SOD, GPx, CAT, and heme-oxygenase-1 (HO-1). Based on these facts, OT may activate Nrf2 via moderate oxidative stress (Sagai & Bocci, 2011).

By increasing the oxidation process, MTX can disrupt the maturation process of sperms, increase the number of immature germ cells, and, as a result, reduce the thickness of the epithelium (Yulug *et al.,* 2013). OT could reverse Johnsen scoring and morphometric parameters, which reflects improving spermatogenesis by the OT in the MTX-treated animals. In another study, ozone alone or in combination with other antioxidants reduced testis damage and preserved spermatid and spermatogonia cells (Salem *et al.,* 2017).

The mice treated with MTX exhibited a reduction in serum testosterone levels. MTX significantly reduces the size of Leydig cells and testosterone production in the testicular tissue (Gutierrez & Hwang, 2017). Leydig cells are very vulnerable to oxidative stress (Awny *et al.,* 2021). Previous studies confirm that oxidative stress induces apoptosis in the Leydig cells (Sun *et al.,* 2017). Hence, the decreased testosterone level by the MTX may result from Leydig cell damage.

Reduced testosterone concentration causes histological changes in the seminiferous tubules (Zeng *et al.,* 2018). Furthermore, a link between declining testosterone levels and inducing apoptosis in germ cells has been established (Rivas *et al.,* 2022). The vacuoles in the germinal epithelium indicate germ cell apoptosis induced by MTX. Previous studies have demonstrated that these vacuoles indicate germ-cell apoptosis (Sönmez *et al.,* 2016; Sultana *et al.,* 2022).

Numerous reports suggest that oxidative stress induces apoptosis, and antioxidants protect against apoptosis in noncancerous cells (Moradi *et al.,* 2023; Sengul *et al.,* 2023; Ijaz *et al.,* 2023). OT could enhance testosterone secretion and decrease vacuolization in the germinal epithelium. Therefore, OT may protect testicular tissue from MTX damage by suppressing germ cell apoptosis. Previous research has demonstrated that OT inhibits apoptosis in response to oxidative stress in diverse tissues, including the kidney, liver, and testis (Mete *et al.,* 2017; Oztosun *et al.,* 2012; Safwat *et al.,* 2014).

In a previous study, OT showed a protective role against ischemia/reperfusion-induced testicular injury by decreasing apoptosis (Aydos *et al.,* 2014).

A limitation of this study was that the relationship between oxidative stress, inflammation, and apoptosis in the experimental groups was not examined. Future studies are needed to evaluate OT impacts on apoptosis and inflammation induced by MTX in the mouse testicles. However, we showed that OT effectively reverses the toxic impacts of MTX on the mouse testicular tissue via enhancing antioxidant activity. Therefore, OT, as an antioxidant, can improve fertility during chemotherapy.

## CONCLUSION

This study has shown that OT reduces the destructive effects of MTX on testicular tissue by activating antioxidant production. In addition, the reversed testosterone level by OT may be due to the androgenic function of Ozone through the preservation of Leydig cells in the MTX-treated mice.
